# Interactive effects of ocean warming and higher irradiances on the Southern Ocean diatom *Pseudo-nitzschia subcurvata*

**DOI:** 10.1007/s00300-026-03542-6

**Published:** 2026-10-10

**Authors:** Olivia Le Gouvello, Ben J. Fisher, Sian F. Henley

**Affiliations:** 1 School of GeoSciences, University of Edinburgh, Edinburgh, UK; 2 Lyell Centre for Earth and Marine Science and Technology, Heriot-Watt University, Edinburgh, UK

**Keywords:** Climate change, Antarctica, Diatoms, Ocean warming, Light availability, Incubation experiments

## Abstract

Diatoms are the dominant primary producers south of the Antarctic Polar Front (~60°S) and play a central role in Southern Ocean food webs and the biological carbon pump. Although models project that climate change will enhance primary productivity in the Southern Ocean, the interactive effects of environmental drivers, particularly temperature and light, on diatom productivity and physiology remain poorly understood. In this study, we investigated the individual and combined effects of ocean warming and increased irradiance on the growth and physiology of the widespread Southern Ocean diatom *Pseudo-nitzschia subcurvata*. We used an experimental matrix consisting of two temperature treatments (2 °C and 8 °C) and two light levels (20 and 200 µmol photons m^−2^ s^−1^), representing current and projected conditions in the Antarctic for 2100 in Coupled Model Intercomparison Project (CMIP) models. The combination of high light and high temperature yielded the highest growth rates and Particulate Organic Carbon (POC) and Nitrogen (PON) production rates. While high light individually enhanced biomass accumulation, growth was significantly higher only when high light and high temperature acted together. Combined high light and high temperature conditions also produced higher-than-expected chlorophyll-to-carbon ratios, and photosynthetic efficiency remained high across treatments. These findings suggest that under future Southern Ocean conditions, diatoms such as *P. subcurvata* may increase their productivity by adjusting their photosynthetic physiology. By quantifying the interactive effects of temperature and light on diatom physiology, this study provides mechanistic insights to productivity changes that can help refine predictions of climate-driven shifts in Southern Ocean primary production.

**Supplementary Information:**

The online version contains supplementary material available at https://doi.org/10.1007/s00300-026-03542-6.

## Introduction

The Southern Ocean plays a crucial role in regulating global climate as a major oceanic sink for carbon dioxide (CO_2_), accounting for approximately 40 % of the ocean’s uptake of anthropogenic CO_2_ (Caldeira and Duffy 2000; DeVries 2014; Frölicher et al. 2015; Mikaloff Fletcher et al. 2006; Orr et al. 2001). The region also supports rich and productive marine ecosystems with unique biodiversity (e.g., David and Saucède 2015; Ducklow et al. 2007; Murphy et al. 2021). Both carbon uptake and biodiversity rely on primary production by marine phytoplankton in the upper ocean (Grigoratou et al. 2025; Sathyendranath and Platt 2001). In the Southern Ocean south of the Antarctic Polar Front (~60°S), primary production is mainly driven by diatoms (Brzezinski et al. 2001), a type of phytoplankton characterised by their relatively large size and shells made of silica (Tréguer et al. 1995). Due to their size, diatoms sink fast and are efficient drivers of the biological carbon pump, removing CO_2_ from the atmosphere and exporting organic carbon to depth where it can be stored for centuries to millennia (Eppley and Peterson 1979). Diatoms are also key to supporting biodiversity in the Southern Ocean as they are the preferred food source for Antarctic krill (Haberman et al. 2003), which in turn provide a critical food source to higher trophic levels, including many marine mammals, fish and seabirds (Krumhardt et al. 2022).

As a result of anthropogenic climate change, Southern Ocean ecosystems are experiencing rapid changes, which vary by latitude, sector and depth. Some regions are experiencing rising seawater temperatures at rates faster than the global average (e.g., Goodell et al. 2024; Meredith et al. 2016) and marine heat waves are becoming more common (Piñones et al. 2024), whilst warming of deep waters is more widespread (Sallée 2018; Schmidtko et al. 2014). Perhaps the most striking change observed in recent years is the dramatic decline in sea ice extent since 2015 (Purich and Doddridge 2023), which has been linked to increasing wind strength and increasing mixed layer depth (Leung et al. 2015), altering light availability across the coastal Southern Ocean (Silvano et al. 2025; Spira et al. 2026). Understanding how phytoplankton are responding to these environmental changes is critical for predicting climate change impacts on Southern Ocean ecosystems (Xue et al. 2024). Evidence shows that climate change is altering the community composition of Southern Ocean phytoplankton (Fisher et al. 2025; Hayward et al. 2025; Mendes et al. 2023). Between 1997 and 2023, sea ice loss was associated with a 33 % decline in diatom abundance and a shift towards smaller phytoplankton groups such as cryptophytes and haptophytes (Hayward et al. 2025), which are more heavily grazed by salps and provide lower nutritional value to higher trophic levels (Mendes et al. 2023). These shifts ultimately weaken food web efficiency and diminish the effectiveness of the biological carbon pump (Deppeler and Davidson 2017).

In addition to phytoplankton community restructuring, climate change is altering the magnitude of primary productivity (Krumhardt et al. 2022). Whereas phytoplankton productivity is expected to decline globally as a result of nutrient limitation (Silsbe et al. 2025), Coupled Model Intercomparison Project (CMIP) models project that primary production is expected to increase as a whole in the Southern Ocean as climate change proceeds (Bopp et al. 2013; Krumhardt et al. 2022; Leung et al. 2015; Moore et al. 2018). However, this increase is not uniform and follows distinct patterns across latitudinal bands corresponding to different circumpolar water masses (Leung et al. 2015). For instance, primary productivity is projected to decrease in the Polar Frontal Zone (between 50°S and 65°S) due to declining light availability linked to a deepening of the summer mixed layer and increased cloud cover and wind strength (Leung et al. 2015). In contrast, the Antarctic Zone south of 65°S is projected to experience enhanced productivity, driven by higher light availability due to declining sea ice cover and shoaling of the mixed layer, and increased supply of iron, a key limiting nutrient essential for light assimilation (Fisher et al. 2025; Krumhardt et al. 2022; Leung et al. 2015). Additionally, concurrent ocean warming is also shifting phytoplankton communities towards their thermal optima which are above present-day temperatures (Thomas et al. 2012). Despite these projections, the physiological mechanisms underpinning productivity changes in phytoplankton communities remain poorly understood.

Controlled incubation studies have elucidated physiological and adaptive strategies of Southern Ocean diatoms in response to single environmental factors (Boyd et al. 2016). Diatoms have higher growth rates when exposed to higher temperatures due to faster metabolic rates (Falkowski and Raven 2013; Raven and Geider 1988). Moreover, photoacclimation is a common physiological response among Southern Ocean phytoplankton, a process through which chlorophyll *a* content is downregulated under higher irradiances to reduce damage by light (Falkowski and LaRoche 1991). However, incubation experiments increasingly show that the physiological processes controlling phytoplankton primary productivity respond in interactive, often non-linear ways to combined environmental stressors (Andrew et al. 2019; Boyd et al. 2016). Previous studies have found, for instance, that increased iron availability combined with higher light intensities enhance species’ ability to tolerate warmer conditions (Andrew et al. 2019), and that higher temperatures improve CO_2_ uptake efficiency in Southern Ocean diatoms (Zhu et al. 2017). Improving our understanding of the mechanisms underlying these interactions is crucial to refine productivity models and provide more accurate estimates of changes in Southern Ocean ecosystems (Boyd et al. 2016). In particular, few culture experiments have focused specifically on disentangling the individual and interactive effects of higher light and increasing temperature on Southern Ocean diatom productivity (Andrew et al. 2019; Boyd et al. 2016).

This study aimed to understand the interactive effects of ocean warming and higher irradiances on Southern Ocean diatom productivity and photosynthetic physiology using *Pseudo-nitzschia subcurvata* as a model organism. This pennate diatom is widely distributed across the Southern Ocean, from sub-Antarctic to Antarctic waters, indicating exposure to a broad range of temperature and light regimes (Bates et al. 2018). This distribution suggests physiological flexibility and makes *P. subcurvata* representative of Antarctic diatoms likely to experience continued warming and enhanced irradiance as surface waters warm, seaice cover declines and growing seasons lengthen (Krumhardt et al. 2022).

Cultures of *P. subcurvata* were grown under different light and temperature treatments representing present and future conditions in the Southern Ocean projected for 2100. Productivity was assessed through growth rate measurements, Particulate Organic Carbon (*POC*) and Particulate Organic Nitrogen (*PON*) production rates, while physiology was evaluated using chlorophyll-to-carbon ratios (*Chl*:*POC*), cell-specific chlorophyll *a* concentrations, and photosynthetic efficiency measured as the maximum quantum yield of photosystem II (${F}_{v}/{F}_{m}$) and non-photochemical quenching (*NPQ*). We aimed to determine the individual and combined effects of higher irradiances and warming on these variables. By quantifying the interactive effects of light and temperature on *P. subcurvata* as a model diatom, we aimed to further our understanding of phytoplankton growth in a future Southern Ocean and improve our ability to represent these interactive effects in model projections.

## Methods

### Experimental set-up

Prior to the main experiment, the diatom *P. subcurvata* (CCMP3560, isolated from the Ross Sea at 75.987° S, 178.9° W, January 2017), was cultured in f/2+Si media for 3 weeks at 2 °C under 100 µmol photons m^−2^ s^−1^ in a light: dark cycle of 18:6 h. The f/2+Si media (Guillard 1975) was made in artificial seawater following Harrison et al. (1980).

The main experiment was conducted using 1-L clear polycarbonate bottles for four experimental treatments, each done in triplicate (*n* = 12). Each bottle contained 75 mL of a *P. subcurvata* starter culture and 925 mL of artificial seawater, enriched with f/2+Si media (Guillard 1975). The experimental treatments comprised two light (20 and 200 µmol photons m^−2^ s^−1^) and two temperature (2 °C and 8 °C) conditions (Table 1). The low temperature (2 °C) was chosen based on previous studies that used it as a reference for current conditions in the Southern Ocean for *P. subcurvata* (Zhu et al. 2017), and the high temperature (8 °C) represents projected conditions in CMIP models under the high emission scenario (SSP5-8.5) for the sub-Antarctic region in 2100 (Bindoff et al. 2019; Krumhardt et al. 2022). The low light intensity (20 µmol photons m^−2^ s^−1^) represented a baseline minimum light level needed for growth, whilst the high light intensity (200 µmol photons m^−2^ s^−1^) reflected potential future irradiances within the Southern Ocean mixed layer (Andrew et al. 2019). In addition to the experimental treatments, two cultures were maintained in complete darkness at 2 °C and 8 °C to act as zero-light controls.

The 14 bottles were distributed between two Panasonic MIR-154 incubators, one set at 2 °C and the other at 8 °C, with temperature variability of ± 2 °C. Bottles exposed to “high light” conditions were placed on the top shelf, where they received 200 µmol photons m^−2^ s^−1^ from full spectrum LED lights, while those in “low light” conditions were placed on the bottom shelf beneath a neutral density screen (LEE Filters) that attenuated light to 20 µmol photons m^−2^ s^−1^. Light levels were checked at each bottle using a PAR sensor (Apogee MQ-500), and the bottles were arranged in a staggered position to avoid light blockage.

Cultures were grown for 11 days in a light:dark cycle of 18:6 h, until sustained growth was observed. The main experiment started with initial cell densities of ~7.4 × 10^5^ cells L^−1^ and ended with cell densities between 43 and 150 × 10^5^ cells L^−1^. Particulate Organic Carbon (*POC*), Particulate Organic Nitrogen (*PON*), and chlorophyll *a* concentrations were measured after 11 days of growth and acclimation to experimental conditions.

**Table 1:** Treatment names with associated light and temperature conditions

Treatment name	Light conditions	Temperature conditions
Low Light Low Temperature	20 µmol photons m^−2^ s^−1^	2 °C
Low Light High Temperature	20 µmol photons m^−2^ s^−1^	8 °C
High Light Low Temperature	200 µmol photons m^−2^ s^−1^	2 °C
High Light High Temperature	200 µmol photons m^−2^ s^−1^	8 °C

### Cell counts

Daily aliquots were taken during the growth period for cell counts (excluding days 5 and 6). After gently inverting the bottles twice, 200 µL of solution from each of the 14 bottles was pipetted onto a welled plate and preserved with 1 % glutaraldehyde. The plates were stored at 4 °C until further analysis. Cell counts were measured from 100-µL acquisitions from each well with a BD LSRFortessa Flow Cytometer. Live cells were identified by size measured on the blue laser (488 nm), and chlorophyll *a* was measured on the red laser (633 nm). Forward Scatter Area (FSC-A) measurements were obtained from Flow Cytometry data and are interpreted as an indirect measure of relative cell size.

### Elemental composition

At the end of the experiment on day 11, cultures were filtered onto pre-combusted and pre-weighed Whatman GF/F 25-mm filters (450 °C for 4 h) with nominal pore size 0.7 μm by vacuum filtration. The amount of solution filtered per bottle depended on the biomass and treatment, and ranged between 80 and 200 mL (Table S1, Online Resource 1). The filters were stored at −20 °C until further analysis. After thawing, filters were fumigated with hydrochloric acid (37 % HCl for 6 h) to remove residual particulate inorganic carbon. Samples were then dried at 40 °C for 12 h and weighed to determine the dry mass. Filters were wrapped in tin capsules (Elemental Microanalysis) and analysed for *POC* and *PON* concentrations using an Elemental Analyzer (Thermo Fisher Flash SMART 2000). Measurements of *POC* and *PON* were corrected for filter blank measurements. Deviation from the certified reference material (BBOT, Elementex) was < 4 % for N and < 1 % for C. *POC* production rates were then calculated as:

$${\rho\:}_{POC}=\frac{{\left[POC\right]}_{t}-\left(\frac{{\left[POC\right]}_{t}}{{N}_{t}}\times\:{N}_{0}\right)}{t}$$

where ${\rho\:}_{POC}$ is the *POC* production rate over the growth phase in µmol L^−1^ day^−1^, ${\left[POC\right]}_{t}$ the cell-specific *POC* content in µmol L^−1^ on the final day of the culture growth phase (day 11), ${N}_{t}$ the cell count at the end of the growth phase, ${N}_{0}$ the cell count at the start of the growth phase (day 7) and *t* the length of the growth phase (4 days). Cell-specific *POC* content was calculated by dividing total *POC* concentrations by final cell counts on day 11. *PON* production rates were calculated with the same equation using *PON* concentrations and *POC*:*PON* was assumed to be constant throughout the growth phase.

### Chlorophyll *a*

At the end of the experiment, between 80 and 200 mL of each culture was filtered onto Whatman GF/F 25-mm filters (pore size ~0.7 μm), varying with culture density (Table S1, Online Resource 1). Filters were stored at −80 °C until further analysis with 10 mL of 90 % acetone to extract the chlorophyll *a* pigment. Chlorophyll *a* analysis was performed using a UV-1900 spectrophotometer and was standardised first with a blank measurement. Following Arar and Collins (1997), absorbance was measured at 664, 647 and 630 nm for each sample. Chlorophyll *a* concentrations were calculated as:

$$\:Chl=11.85\left({Abs}_{664}\right)-1.54\left({Abs}_{647}\right)-0.08\left({Abs}_{630}\right)$$

where *Chl* is the concentration of chlorophyll *a*, ${Abs}_{644}$ is the absorbance measured at 664 nm, ${Abs}_{647}$ is the absorbance measured at 647 nm and ${Abs}_{630}$ is the absorbance measured at 630 nm.

### Photophysiology

Photosynthetic efficiency can be measured through the maximum quantum yield of photosystem II (${F}_{v}/{F}_{m}$) and non-photochemical quenching (*NPQ*). ${F}_{v}/{F}_{m}$ is a measure of the efficiency of light energy conversion into fixed carbon and *NPQ* is a photoprotective mechanism through which excess absorbed light is dissipated as heat in photosystem II (PSII) (Giovagnetti and Ruban 2015). High photosynthetic efficiency therefore is reflected by high ${F}_{v}/{F}_{m}$ and low *NPQ* rates.

Chlorophyll *a* fluorescence was determined from 2-mL aliquots taken daily during the growth period (excluding days 5 and 6) after gently inverting each bottle twice. Measurements were performed with a Fast Repetition Rate Fluorometer (FRRF) and a FastAct Laboratory system (Chelsea Technologies Group Ltd), and values were obtained using the FastPro8 software. Samples were first dark-acclimated under red light in an incubator at 2 °C for 30 min to allow PSII reaction centres to reach a stable state (Pagnone et al. 2021). The measurement settings were pre-determined using a small sample from the starter culture and optimised specifically for *P. subcurvata*. Samples were kept in dark-acclimated conditions throughout the measurement period.

${F}_{v}/{F}_{m}$, the maximum quantum yield of PSII, was calculated by the FastPro8 software as:

$${F}_{v}/{F}_{m}=\left({F}_{m}-{F}_{0}\right)/{F}_{m}$$

where ${F}_{0}$ is the minimum chlorophyll *a* fluorescence after dark acclimation before exposure to light, and ${F}_{m}$ is the maximum fluorescence measured after dark acclimation during light saturation. For each sample, the ${F}_{v}/{F}_{m}$ value was determined by averaging the two ${F}_{v}/{F}_{m}$ measurements obtained at PAR = 0 µmol photons m^−2^ s^−1^.

*NPQ* was determined with the following formula:

$$NPQ=({F}_{m}-\:{F}_{{m}^{{\prime}}})/{F}_{{m}^{{\prime}}}$$

where ${F}_{m}$ is the average maximum fluorescence level obtained from two measurements taken under PAR = 0 µmol photons m^−2^ s^−1^, and ${F}_{{m}^{{\prime}}}$ is the average light-adapted maximum fluorescence level obtained from two measurements taken under PAR = 1219 µmol photons m^−2^ s^−1^. Fluorescence parameters (${F}_{m}$ and ${F}_{{m}^{{\prime}}}$) were normalised to biomass to account for differences in cell density among treatments.

The FRRF “chlorophyll” value was interpreted as a relative measure of optical density and was monitored daily as an additional proxy for growth alongside cell numbers.

### Growth rate metrics

Growth rates were measured with two metrics, each with strengths and limitations, to obtain a comprehensive estimate of growth. For both metrics, growth rates were measured for the growth phase, identified as occurring between days 7 and 11 based on plots of cell counts over time (Fig. S1, Online Resource 1).

The first growth metric was specific growth rate (*μ*), calculated as

$$\mu\:=\:\mathrm{l}\mathrm{n}({N}_{t}/{N}_{0})/t$$

where ${N}_{t}$ are cell counts on day 11, ${N}_{0}$ are cell counts on day 7, and *t* is the length of the growth period (4 days) after the stationary phase. While this method provided a direct measure of growth rates, daily cell counts were measured from small volumes (100 µL) relative to the bottle volume (1 L), leading to variability between samples within the same treatment.

The second growth metric was optical density measured by the FRRF, calculated as:

$$\:optical\:density=\mathrm{l}\mathrm{n}({Chl}_{t}/{Chl}_{0})/t$$

where ${Chl}_{t}$ is the absorbance of chlorophyll *a* measured on day 11, and ${Chl}_{0}$ is the absorbance of chlorophyll *a* measured on day 7. The rate of optical density change was given in Arbitrary Units per day (A.U. day^−1^). The advantage of using this metric was that the sampled volume was larger than for cell counts (2 mL), which increased precision. However, optical density served as a growth proxy rather than a direct measurement of growth.

### Statistical analysis

All statistical analyses conducted in R (version 4.2.1) using RStudio (version 2023.9.1.494). To determine the individual and combined effects of light (20 and 200 µmol photons m^−2^ s^−1^) and temperature (2 °C and 8 °C) on parameters among different treatments of *P. subcurvata*, t-tests and 2-way ANOVAs followed by Tukey–Kramer post-hoc tests were used. Assumptions of normality of residuals and homoscedasticity (equal variances) required for parametric analyses were evaluated using Shapiro and Bartlett tests, respectively. Log transformations were applied when raw data violated assumptions of normality or homoscedasticity, or when analyses on log-transformed values better reflected the observed patterns in the data. A noticeable outlier in the high light 2 °C treatment (sample 4) was excluded from chlorophyll concentration measurements due to the abnormally low value suggesting a measurement error during the analysis.

## Results

### Growth rates

**Fig. 1:**
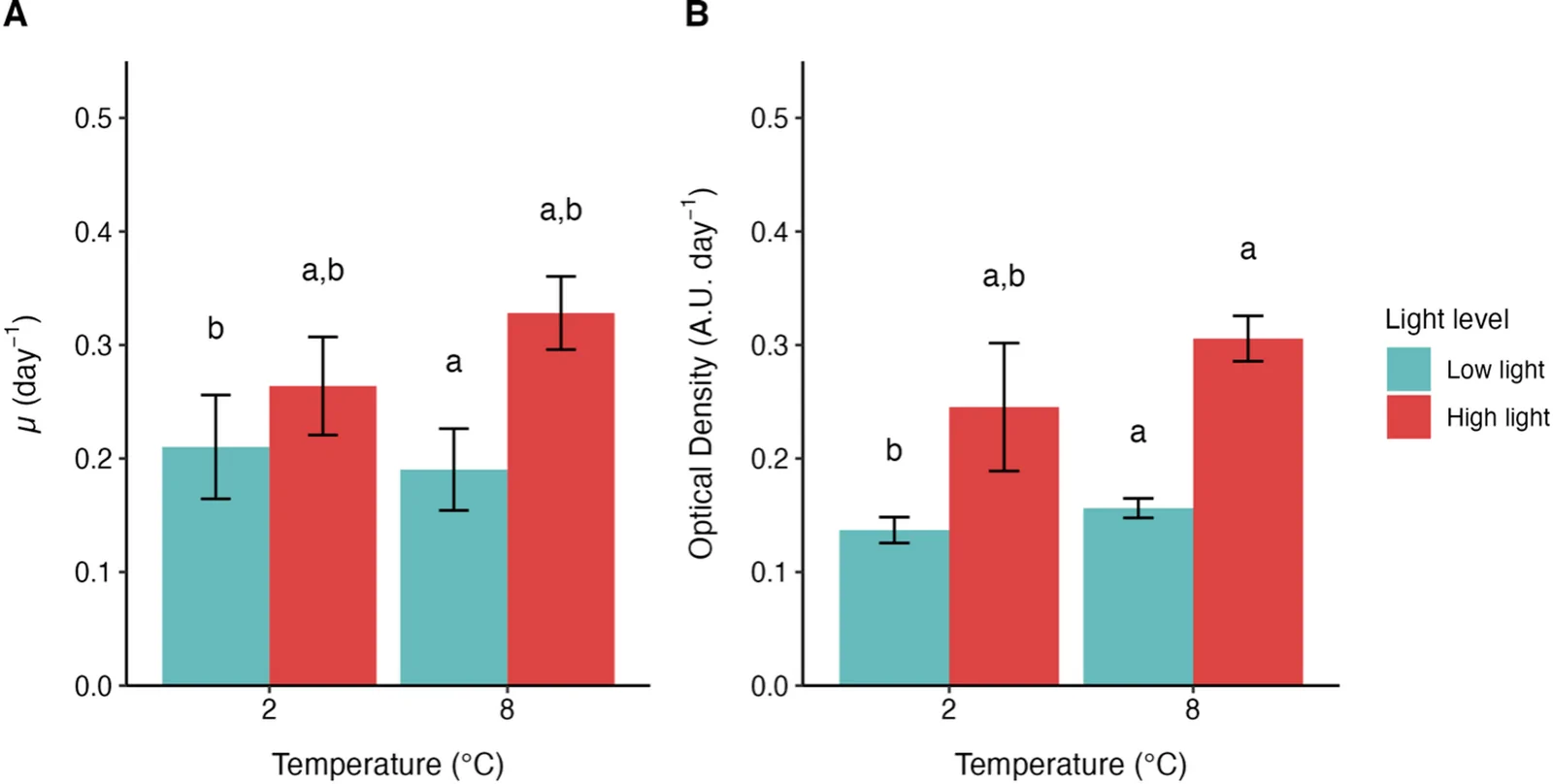
Specific growth rates ($\:\mu\:$) (day^−1^) (**A**) and rates of change in optical density (A.U. day^−1^) (**B**) for each treatment between the start and end of the growth phase (days 7–11). Error bars represent standard errors of the means for each treatment (*n* = 3) and letters indicate significant differences between treatments (i.e., a is significantly different to b, whilst a, b is not statistically different from either a or b)

Treatments started with initial cell densities of ~7.4 × 10^5^ cells L^−1^ and ended with cell densities between 43 and 150 × 10^5^ cells L^−1^ (Fig. S1, Online Resource 1). The highest growth rates for *P. subcurvata*, measured both through specific growth rate (*μ*) and daily change in optical density, were observed at 8 °C under high light (*μ*: 0.33 ± 0.03 day^−1^ and optical density: 0.31 ± 0.02 A.U. day^−1^; Fig. 1). These rates were significantly higher compared to growth rates at 8 °C under low light, both for *μ* (0.19 ± 0.04 day^−1^, Welch’s two-sample t-test, *t*_(3.95)_ = −2.85, *p* = 0.0470; Fig. 1A) and optical density (0.16 ± 0.01 A.U. day^−1^, Tukey–Kramer, *p* = 0.0360; Fig. 1B). A significant light effect was confirmed by 2-way ANOVA (*μ*: *F*_1,8_ = 5.83, *p* = 0.0422; optical density: *F*_1,8_ = 17.58, *p* = 0.0030; Fig. 1). In contrast, no difference was observed at 2 °C between high and low light treatments (*μ*: Welch’s two-sample t-test, *t*_(3.99)_ = −0.85, *p* = 0.442; optical density: Tukey–Kramer, *p* = 0.969; Fig. 1). Temperature did not have a significant effect on growth rates (2-way ANOVA, *μ*: *F*_1,8_ = 0.31, *p* = 0.591; optical density: *F*_1,8_ = 1.68, *p* = 0.231; Fig. 1) and no significant interaction term between light and temperature was found (2-way ANOVA, *μ*: *F*_1,8_ = 1.13, *p* = 0.320; optical density: *F*_1,8_ = 0.45, *p* = 0.523; Fig. 1). Growth rates under high light and 8 °C were significantly higher than under low light and 2 °C for optical density (Tukey–Kramer, *p* = 0.0196). Specific growth rates also showed similar trends (*μ*: 0.33 ± 0.03 day^−1^ vs. 0.19 ± 0.04 day^−1^), but this difference was not significant (Welch’s two-sample t-test, *t*_(3.59)_ = −2.11, *p* = 0.111; Fig. 1A).

### Particulate organic carbon (*POC*) and particulate organic nitrogen (*PON*) production rates

**Fig. 2:**
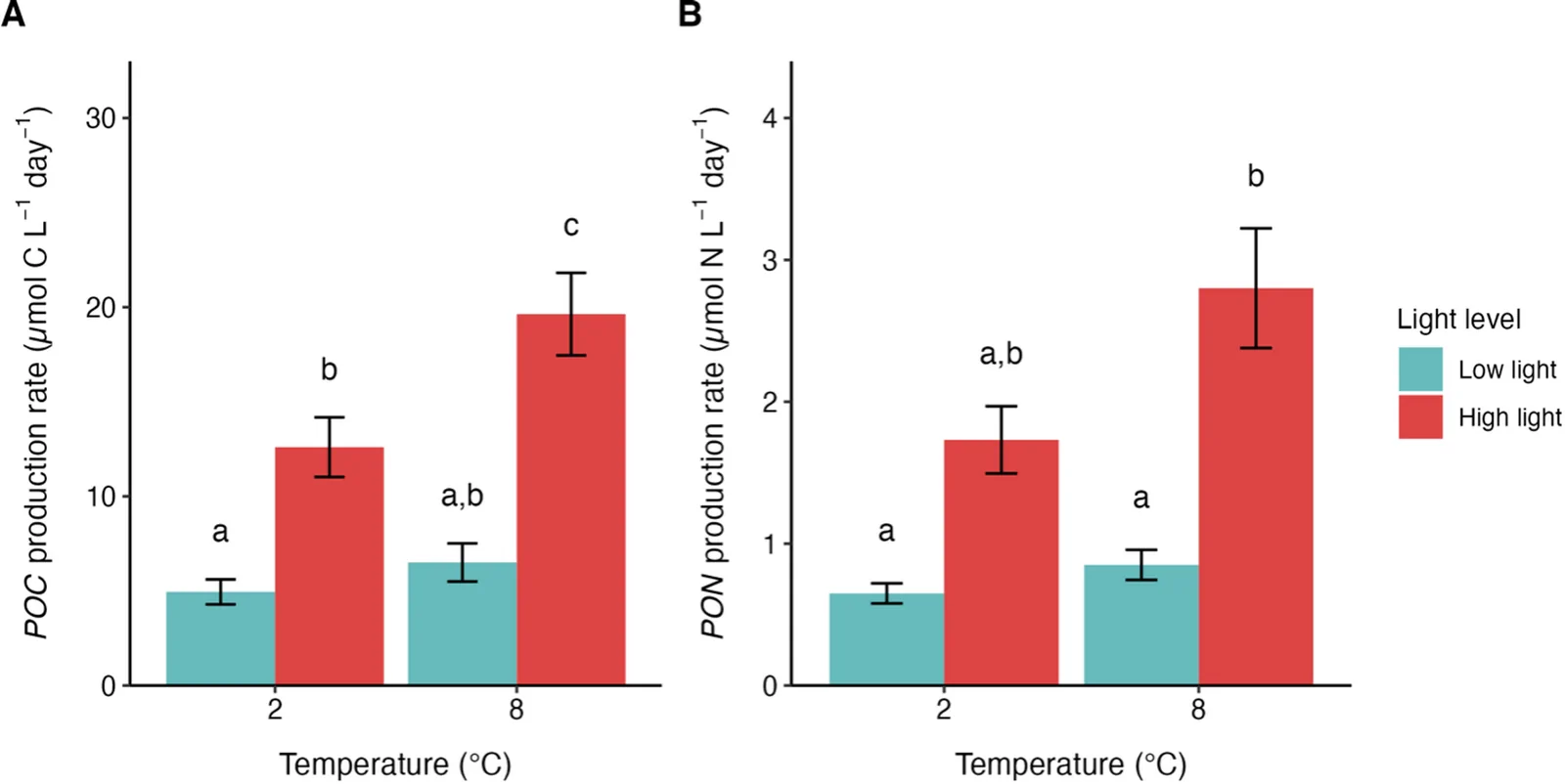
Production rates of Particulate Organic Carbon (* POC*) (µmol C L^−1^ day^−1^) (**A**), and Particulate Organic Nitrogen (* PON*) (µmol N L^−1^ day^−1^) (**B**) calculated over the growth phase (days 7–11). Error bars are the standard errors of the means (*n* = 3) and letters indicate significant differences between treatments (i.e., a, b, and c are all significantly different from each other, whilst a, b is not statistically different from either a or b)

The highest *POC* and *PON* production rates were observed under high light and 8 °C (19.60 ± 2.18 µmol C L^−1^ day^−1^ and 2.80 ± 0.42 µmol N L^−1^ day^−1^, respectively), while the lowest *POC* and *PON* production rates were in the low light and 2 °C treatment (4.95 ± 0.66 µmol C L^−1^ day^−1^ and 0.65 ± 0.07 µmol N L^−1^ day^−1^ respectively). Light had a significant effect on both *POC* production rates (2-way ANOVA, *F*_1,8_ = 49.63, *p* = 0.0001; Fig. 2A) and *PON* production rates (2-way ANOVA, *F*_1,8_ = 36.74, *p* = 0.0003; Fig. 2B). At both 2 °C and 8 °C, high light consistently resulted in higher *POC* production rates than low light (Tukey–Kramer: *p* = 0.0261 at 2 °C and *p* = 0.0011 at 8 °C). *PON* production rates were also higher under high light than under low light at 2 °C (1.73 ± 0.24 µmol N L^−1^ day^−1^ vs. 0.65 ± 0.07 µmol N L^−1^ day^−1^) as well as at 8 °C (Tukey–Kramer: *p* = 0.0025). Moreover, temperature had a significant effect on *POC* production rates (2-way ANOVA, *F*_1,8_ = 8.48, *p* = 0.0195; Fig. 2A), with higher values at 8 °C than at 2 °C under high light (Tukey–Kramer: *p* = 0.0394), and no significant difference under low light (Tukey–Kramer: *p* = 0.876). For *PON* production rates, the effect of temperature was significant (2-way ANOVA, *F*_1,8_ = 6.45, *p* = 0.0348; Fig. 2B), and *PON* production rates were higher at 8 °C than at 2 °C under high light (2.80 ± 0.42 µmol N L^−1^ day^−1^ vs. 1.73 ± 0.24 µmol N L^−1^ day^−1^), but not under low light (Tukey–Kramer: *p* = 0.939). There was no significant interaction term for *POC* and *PON* production rates between light and temperature (2-way ANOVA: *F*_1,8_ = 3.45, *p* = 0.100 for *POC*, *F*_1,8_ = 3.01, *p* = 0.121 for *PON*; Fig. 2A and B). *POC*:*PON* ratios remained stable across treatments (ranging from 7.09 ± 0.29 to 7.60 ± 0.31; Table 2), and cell-specific *POC* did not significantly differ between treatments (ranging from 86.3 ± 11.0 to 100.1 ± 3.03 pg cell^−1^; Table 2). Finally, FSC-A measurements were significantly higher under high light at both temperatures (Tukey–Kramer: *p* = 0.0004 at 2 °C, *p* < 0.0001 at 8 °C; Fig. S2, Online Resource 1). FSC-A was significantly higher at 8 °C than at 2 °C under high light (Tukey–Kramer: *p* = 0.0175), but showed no difference under low light (Tukey–Kramer: *p* = 0.799).

**Table 2:** Total Particulate Organic Carbon (*POC*) and Particulate Organic Nitrogen (*PON*) concentrations (µmol L^−1^), *POC*:*PON*, total chlorophyll *a* (*Chl*) concentrations (µg L^−1^) and cell-specific *POC* for each treatment (pg cell^−1^). Errors are standard deviations for *POC*, *PON* and *Chl* concentrations (*n* = 3), and standard errors for *POC*:*PON* and cell-specific *POC* (*n* = 3). Different letters indicate significant differences between treatments

Parameter	Treatments			
	2 °C		8 °C	
	Low Light	High Light	Low Light	High Light
*POC *concentrations (µmol L^−1^)	35.9 ± 2.7 ^a^	78.4 ± 5.4 ^b^	49.5 ± 2.1 ^c^	108.0 ± 18.5 ^d^
*PON *concentrations (µmol L^−1^)	4.7 ± 0.4 ^a^	10.7 ± 1.0 ^b^	6.5 ± 0.2 ^a^	15.4 ± 3.8 ^b^
*POC*:*PON*	7.6 ± 0.2 ^a^	7.3 ± 0.1 ^a^	7.6 ± 0.3 ^a^	7.1 ± 0.3 ^a^
Total *Chl *(µg L^−1^)	23.2 ± 8.7 ^a^	8.1 ± 1.6 ^b^	24.1 ± 8.0 ^a^	20.5 ± 1.4 ^a^
Cell-specific *POC* (pg cell^−1^)	86.3 ± 11.0 ^a^	87.1 ± 11.6 ^a^	100.1 ± 3.03 ^a^	93.9 ± 4.65 ^a^

### Chlorophyll *a* (*Chl*) concentrations, *Chl*: *POC *and cell-specific *Chl*

Starting from initial cell densities of ~7.4 × 10^5^ cells L^−1^, treatments developed diverging total *Chl* concentrations by the end of the growth period. The low light treatments had the highest total *Chl* concentrations (23.2 ± 8.7 µg L^−1^ at 2 °C, 24.1 ± 8.0 µg L^−1^ at 8 °C), and both treatments presented large within-treatment variability. The lowest total *Chl* concentrations were found under high light and 2 °C (8.1 ± 1.6 µg L^−1^). Light had a significant effect on *Chl* concentrations (2-way ANOVA, *F*_1,7_ = 6.91, *p* = 0.0340; Table 2). At 2 °C, *Chl* concentrations were significantly higher under low light than under high light (Tukey–Kramer, *p* = 0.0308), while there was no significant difference between treatments at 8 °C (Tukey–Kramer, *p* = 0.947). Temperature also significantly influenced *Chl* concentrations (2-way ANOVA, *F*_1,7_ = 6.08, *p* = 0.0431; Table 2). Under high light, *Chl* concentrations were significantly higher at 8 °C than at 2 °C (Tukey–Kramer, *p* = 0.0430) while no difference was found between treatments under low light (Tukey–Kramer, *p* = 0.994). Since light and temperature effects were interdependent, there was a significant interaction term between light and temperature for *Chl* concentrations (2-way ANOVA, *F*_1,7_ = 5.72, *p* = 0.0480).

**Fig. 3:**
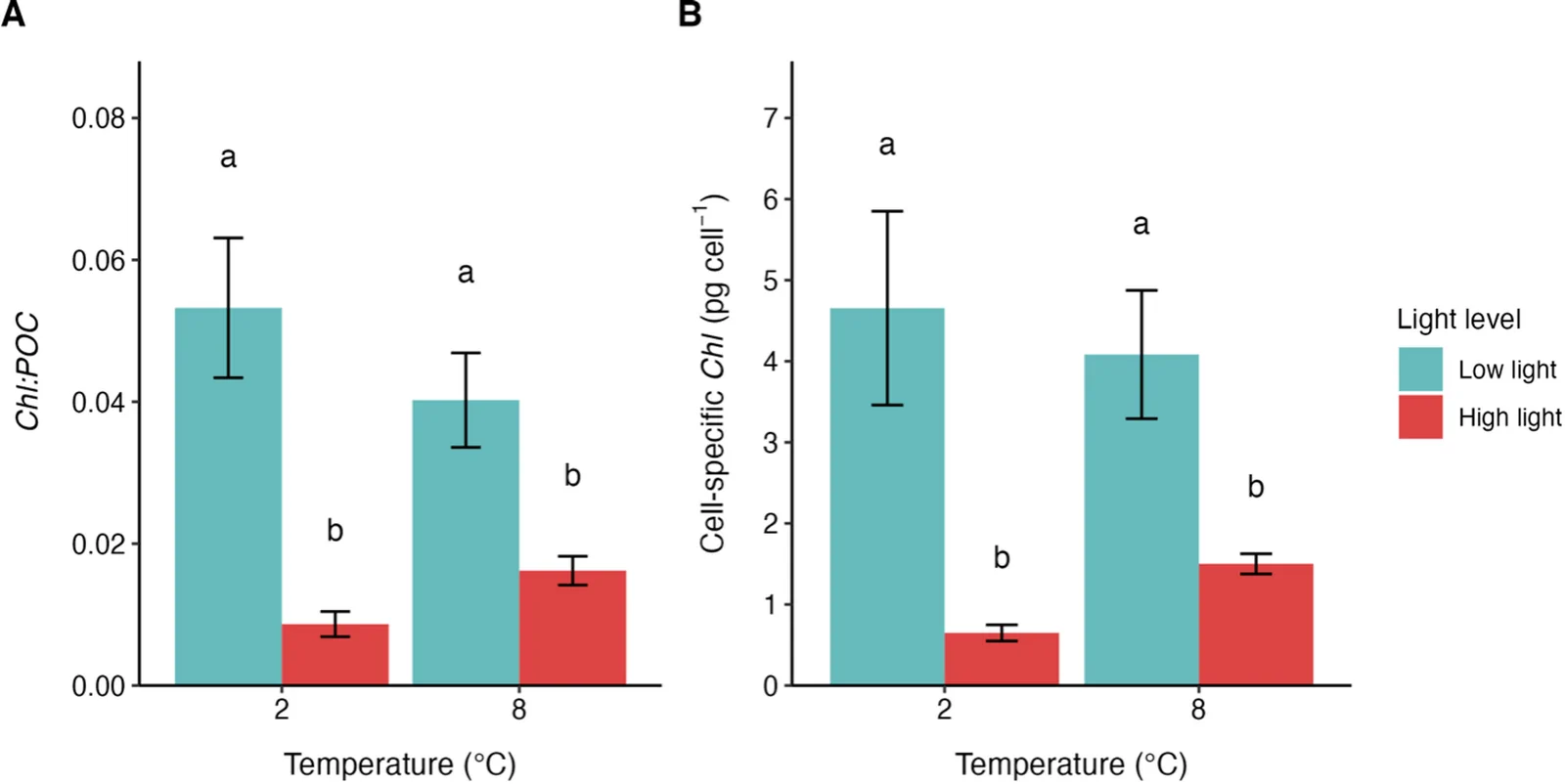
Mean chlorophyll-to-carbon ratio (* Chl*:* POC)* (**A**) and cell-specific chlorophyll (* Chl*) (pg cell^−1^) (**B**) for each treatment. Error bars are the standard errors of the means (*n* = 3) and different letters indicate significant differences between treatments

Light had a significant effect on *Chl*:*POC* (2-way ANOVA, *F*_1,7_ = 51.98, *p* = 0.0002; Fig. 3A). *Chl*:*POC* was consistently lower under high light than under low light at both temperatures (Tukey–Kramer, *p* = 0.0012 at 2 °C, *p* = 0.0286 at 8 °C). The reduction in *Chl*:*POC* under high light was smaller at 8 °C than at 2 °C (60 % vs. 84 % decrease). Temperature, on the other hand, had no significant effect on *Chl*:*POC* (2-way ANOVA, *F*_1,7_ = 0.55, *p* = 0.483; Fig. 3A). However, *Chl*:*POC* was higher at 8 °C than at 2 °C under high light (0.016 ± 0.002 vs. 0.009 ± 0.002). Treatments showed no difference in *Chl*:*POC* between low and high temperature under low light conditions (0.053 ± 0.010 at 2 °C, 0.040 ± 0.007 at 8 °C; Tukey–Kramer, *p* = 0.701). The interaction term between light and temperature for *Chl*:*POC* was significant (2-way ANOVA, *F*_1,7_ = 6.14, *p* = 0.0423; Fig. 3A).

Light had a significant effect on cell-specific *Chl* (2-way ANOVA, *F*_1,7_ = 49.32, *p* = 0.0002; Fig. 3B). At both temperature conditions, cells grown under high light had a lower cell-specific *Chl* concentration than those grown under low light (86 % lower at 2 °C, Tukey–Kramer, *p* = 0.0014; 63 % lower at 8 °C, Tukey–Kramer, *p* = 0.0293). While temperature had no statistically significant effect on cell-specific *Chl* (2-way ANOVA, *F*_1,7_ = 2.73, *p* = 0.143; Fig. 3B), cell-specific *Chl* was higher at 8 °C than at 2 °C under high light (1.50 ± 0.125 pg cell^−1^ vs. 0.649 ± 0.100 pg cell^−1^). Cell-specific *Chl* showed no significant difference between low and high temperature under low light (4.65 ± 1.20 pg cell^−1^ at 2 °C, 4.08 ± 0.791 pg cell^−1^ at 8 °C; Tukey–Kramer, *p* = 0.981). Cell-specific *Chl* was also significantly higher under low light and 2 °C than under high light and 8 °C (Tukey–Kramer, *p* = 0.0186). There was a significant interaction term between light and temperature for cell-specific *Chl* (2-way ANOVA, *F*_1,7_ = 5.76, *p* = 0.0474; Fig. 3B).

### Photosynthetic efficiency

**Fig. 4:**
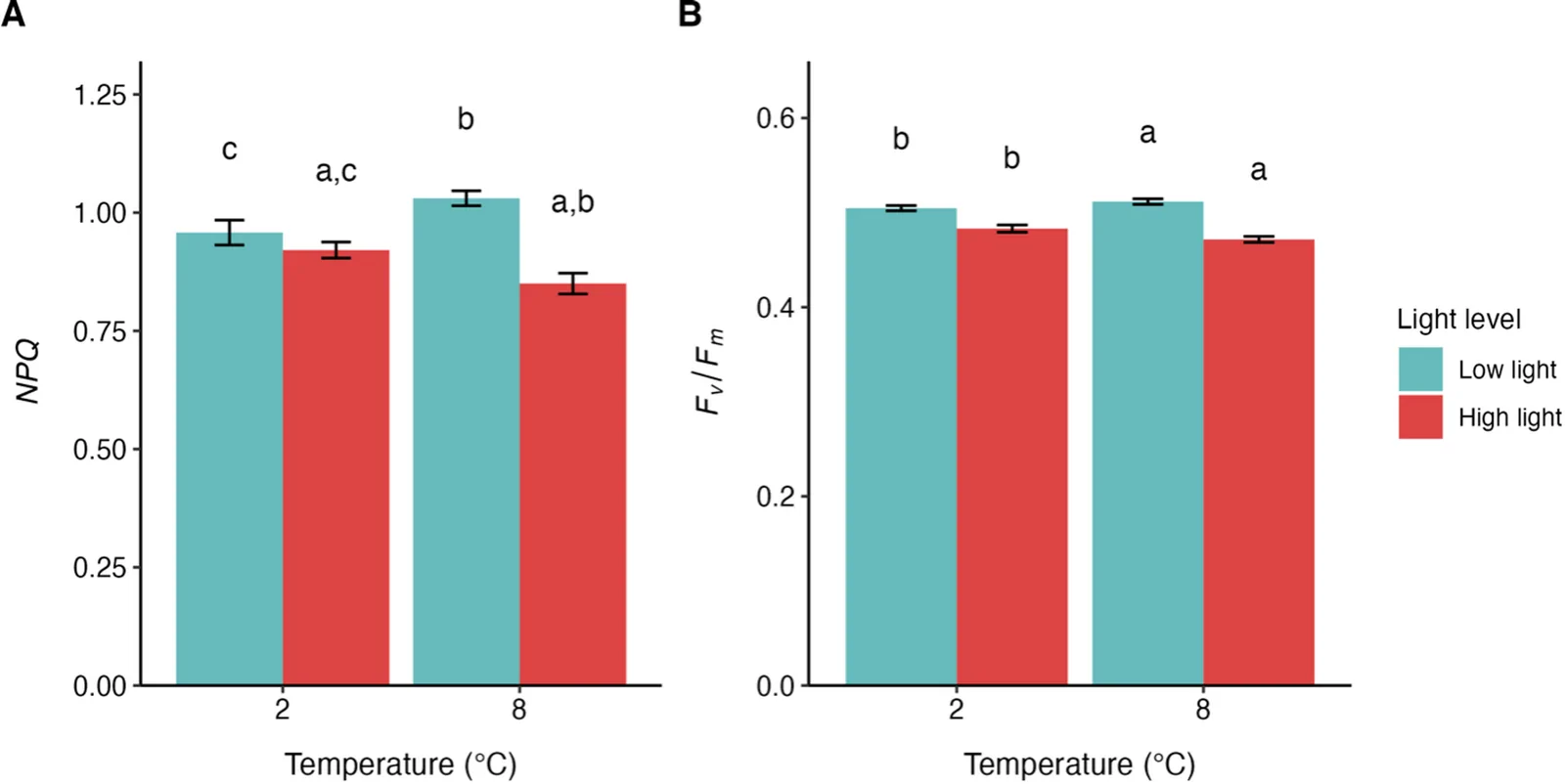
Mean non-photochemical quenching (* NPQ)* (**A**) and maximum quantum yield of photosystem II ($\:{F}_{v}/{F}_{m})$ (**B**) in treatments during the growth phase (days 7–11). Error bars are standard errors of the means (*n* = 15) and different letters indicate significant differences between treatments

*NPQ* ranged between 0.85 ± 0.02 (*n* = 15) (high light, high temperature), and 1.03 ± 0.02 (*n* = 15) (low light, high temperature). Light had a significant effect on *NPQ* (2-way ANOVA, *F*_1,55_ = 26.52, *p* < 0.0001; Fig. 4A). At 8 °C, *NPQ* was significantly higher under low light than under high light (Tukey–Kramer, *p* < 0.0001), whereas no difference was observed at 2 °C (Tukey–Kramer, *p* = 0.589). Temperature had no effect on *NPQ* (2-way ANOVA, *F*_1,55_ = 0.01, *p* = 0.920; Fig. 4A), but there was a significant interaction term between light and temperature for *NPQ* (2-way ANOVA, *F*_1,55_ = 11.79, *p* = 0.0011; Fig. 4A).

${F}_{v}/{F}_{m}$ values ranged from 0.472 ± 0.003 (*n* = 15) to 0.512 ± 0.003 (*n* = 15). The maximum quantum yield of PSII for *P. subcurvata* was significantly affected by light (2-way ANOVA, *F*_1,55_ = 89.68, *p* < 0.0001; Fig. 4B), with values consistently lower under high light compared to low light (Tukey–Kramer: *p* < 0.0001 at 2 °C, *p* < 0.0001 at 8 °C). Temperature did not have a significant effect on ${F}_{v}/{F}_{m}$ (2-way ANOVA, *F*_1,55_ = 0.41, *p* = 0.525; Fig. 4B). Pairwise comparisons further showed that ${F}_{v}/{F}_{m}$ was significantly lower under high light and 8 °C compared to low light and 2 °C (Tukey–Kramer, *p* < 0.0001), and under high light and 2 °C compared to low light and 8 °C (Tukey–Kramer, *p* < 0.0001).

## Discussion

**Fig. 5:**
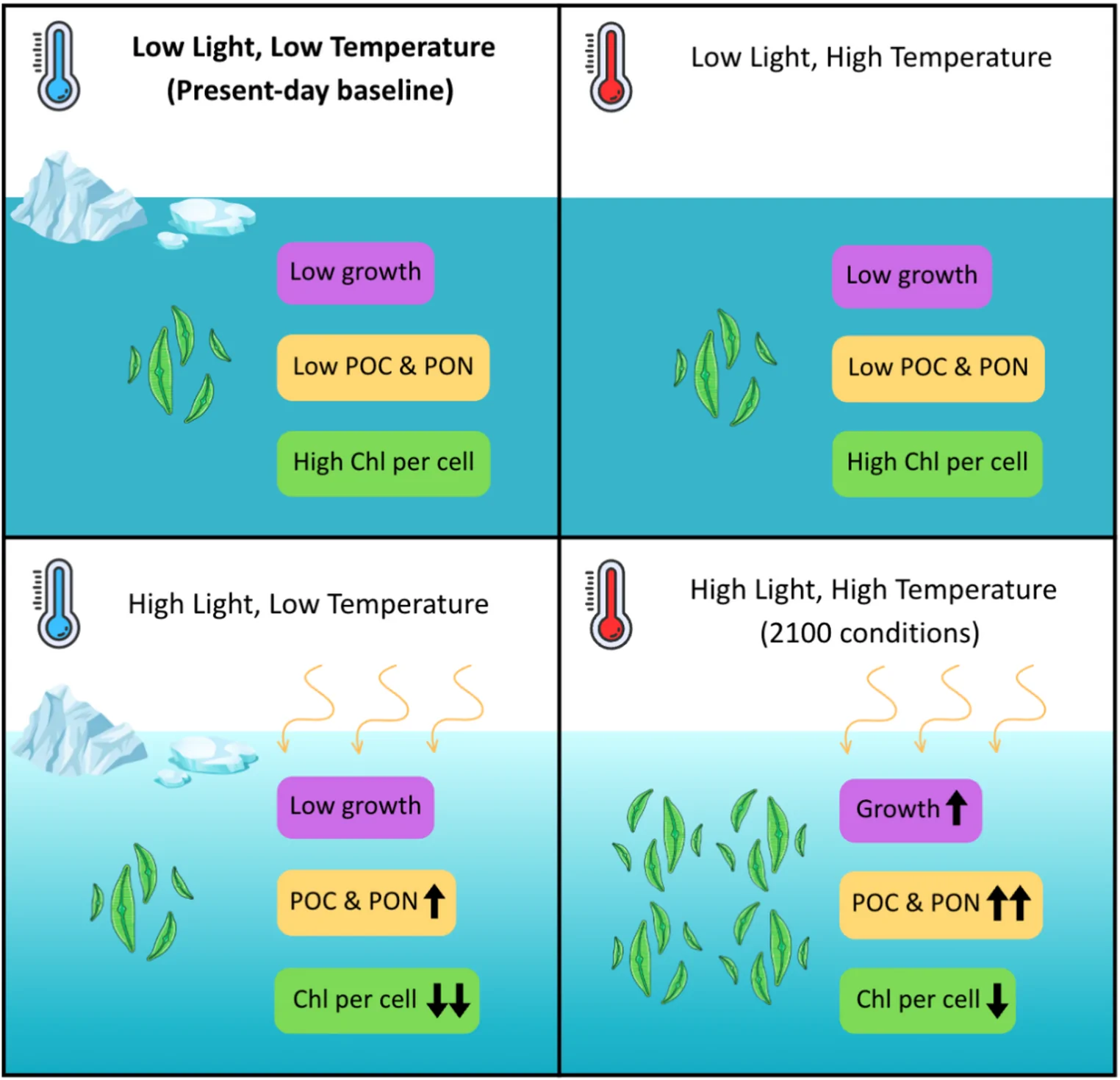
Schematic diagram showing productivity and physiology responses of *Pseudo-nitzschia subcurvata* to light and temperature treatments relative to present-day conditions in the Southern Ocean. Low light refers to 20 µmol photons m^−2^ s^−1^ and high light refers to 200 µmol photons m^−2^ s^−1^, whilst low temperature refers to 2 °C and high temperature refers to 8 °C

Diatoms are central in regulating biogeochemical cycles and sustaining biodiversity in the Southern Ocean (Brzezinski et al. 2001; Eppley and Peterson 1979; Krumhardt et al. 2022). Understanding their response to co-occurring environmental drivers is critical in predicting the impact of climate change on Southern Ocean ecosystems (Boyd et al. 2016; Xu et al. 2014). This study examined the individual and combined effects of ocean warming and higher irradiances on the productivity and physiology of the Southern Ocean diatom *P. subcurvata*. Results show that the combination of high temperatures and high irradiances yielded the highest growth rates and production rates of both *POC* and *PON* (Fig. 5). While *POC* and *PON* production rates consistently increased in response to the individual effect of high light, growth rates rose significantly only under their combined influence (Fig. 5). Moreover, the combination of both factors produced significant temperature-light interactions for *Chl*:*POC* and cell-specific *Chl* (Fig. 5), while *NPQ* and ${F}_{v}/{F}_{m}$ decreased with higher irradiances.

### Interactive effects of light and temperature on *P. subcurvata* productivity

Cultures of *P. subcurvata* grown under the combination of high light (200 µmol photons m^−2^ s^−1^) and high temperature (8 °C), which are conditions representative of future Antarctic scenarios in 2100 (Bindoff et al. 2019), showed the greatest productivity of all treatments, with the highest growth rates (both specific growth and optical density) and *POC* and *PON* production rates (Figs. 1 and 2). Growth rates reached 0.33 ± 0.03 day^−1^ for *μ* and 0.31 ± 0.02 A.U. day^−1^ for optical density, which is higher than the 0.22 day^−1^ average reported for small Southern Ocean diatoms in an in-situ study under similar off-shelf conditions (Irion et al. 2021). In these future scenario conditions, rates of change in optical density were two times higher than at low light (20 µmol photons m^−2^ s^−1^) and low temperature (2 °C), which represent the present-day conditions (Fig. 1B). Similarly, *POC* and * PON* production rates were 3 times higher in high light, high temperature conditions than in low light, low temperature conditions (Fig. 2). These findings align with current model predictions that primary production in the Southern Ocean will increase with climate change, due to ocean warming and enhanced light exposure with increased surface stratification and longer ice-free periods (Bopp et al. 2013; Krumhardt et al. 2022; Leung et al. 2015; Moore et al. 2018). The study also revealed important interactions between light and temperature with regard to productivity. At 2 °C, elevated irradiance did not stimulate growth, whereas at 8 °C, higher light significantly enhanced growth, with approximately a two-fold increase (Fig. 1A and B). While the effect of light was modulated by temperature for growth, elevated irradiances consistently increased *POC* and *PON* production rates at both temperatures, with 1.5 to 2-fold increases (Fig. 2). Stable *POC*:*PON* ratios (Table 2) consistent with the Redfield ratio (C:N = 106:16) indicate that the increase in *POC* and *PON* production rates resulted from a balanced biomass accumulation rather than disproportionate storage of carbon or nitrogen arising from nutrient limitation (Spilling et al. 2015). The increase in productivity under high light conditions is consistent with *P. subcurvata* light curves showing that electron transport rates (ETR) increase with higher light intensities for cultures grown at 2 °C, before decreasing at around 500 µmol photons m^−2^ s^−1^ due to photoinhibition (Camoying et al. 2022). Our findings suggest that while light drove photosynthetic activity and enhanced carbon and nitrogen accumulation, only in combination with higher temperatures did this additional energy translate into increased growth rates, suggesting a co-dependent interaction. This could be explained by higher metabolic rates associated with warmer temperatures (Falkowski and Raven 2013; Raven and Geider 1988). Primary production models that overlook this light–temperature interaction may therefore underestimate future productivity.

Higher temperatures also led to an increase in productivity, but only under high light conditions, as indicated by the higher *POC* and *PON* production rates at 8 °C than at 2 °C (Fig. 2A and B). This effect is consistent with enhanced metabolic rates and carbon fixation at higher temperatures (Falkowski and Raven 2013; Raven and Geider 1988) although it was only apparent in our study when photon availability was non-limiting. However, temperature individually had no effect on growth rates (from either cell counts or optical density) (Fig. 1A and B). The absence of a temperature effect on growth rates differs from previous studies that identify temperature as a major growth driver for Southern Ocean phytoplankton (Boyd et al. 2016; Fiala and Oriol 1990; Jabre et al. 2021), reflecting that polar phytoplankton have thermal optima above current environmental conditions. Temperature was found to increase the growth of *P. subcurvata* in another incubation study, with higher growth rates reported at 8 °C compared to 2 °C (0.88 vs. 0.60 day^−1^) under irradiance of 80 µmol photons m^−2^ s^−1^, and 8 °C being the optimum growth temperature (Zhu et al. 2017). Differences in growth rates between Zhu et al. (2017) and our experiment may partly be due to strain origin, as Zhu et al. (2017) used an Antarctic, ice-edge strain of *P. subcurvata* rather than the sub-Antarctic strain used here, and meltwater-associated conditions have been reported as most favorable for growth of this species (Petrou et al. 2011). The decoupling between biomass accumulation and growth rates may instead reflect an increase in cell size. Relative cell sizes inferred from FSC-A were greater at 8 °C than at 2 °C under high light conditions (Fig. S2, Online Resource 1), mirroring *POC* and *PON* production rates. This suggests that the enhanced *POC* and *PON* production resulted primarily from larger cells rather than increased rates of cell division, similar to responses observed in other small diatoms such as *Thalassiosira pseudonana* (Zhao et al. 2025).

### Interactive effects of light and temperature on the photosynthetic physiology of *P. subcurvata*

Light and temperature had interactive effects on the photosynthetic physiology of *P. subcurvata*. At 2 °C, higher irradiances decreased *Chl*:*POC* ratios (Fig. 3A), due to *POC* accumulation combined with a reduction in *Chl* concentrations from declining cell-specific *Chl* (Figs. 2A and 3B; Table 2). This pattern is consistent with photoacclimation responses observed in *P. subcurvata* (Kulk et al. 2019) and other phytoplankton (Falkowski and LaRoche 1991), where cells induce chlorophyll downregulation to reduce light absorption and mitigate photo-damage. Temperature influenced this light-driven response in our study: declines in *Chl*:*POC* were less pronounced at 8 °C than at 2 °C (60 % vs. 84 % downregulation, Fig. 3B), indicating that higher temperatures mitigated *Chl* downregulation. This mitigation effect therefore enabled cells to increase their light absorption capacity at higher temperatures. These trends were clear, despite particularly high chlorophyll variability under low light conditions, which was likely due to low cell densities (Fig. S1, Online Resource 1).

Light also influenced the effect of temperature on *Chl* production. While *Chl*:*POC* ratios were stable in response to warming under low light, they increased with warming under high light (Fig. 3A). The trend under high light was due to a disproportionate increase in *Chl* relative to *POC* concentrations resulting from increased cell-specific *Chl* under higher temperatures (Fig. 3B). Similar responses have been observed in other *Pseudo-nitzschia* species (Boyd et al. 2016; El-Sabaawi and Harrison 2006): increased *Chl* content with warming likely enables cells to enhance light capture ability to meet the heightened metabolic demand from warmer temperatures (Falkowski and Raven 2013). However, this response was evident in our study only when photon supply was abundant, suggesting that temperature effects on chlorophyll allocation for *P. subcurvata* are light-dependent.

Together, the mitigation of light-driven chlorophyll downregulation by warming, combined with the warming-induced increase in *Chl* production under high light, resulted in relatively high cell-specific *Chl* in the high light and high temperature treatment (Fig. 3B). Both *Chl*:*POC* and cell-specific *Chl* presented significant terms for light and temperature interactions in 2-way ANOVA tests (*Chl*:*POC*: *F*_1,7_ = 6.14, *p* = 0.0423, Fig. 3A & cell-specific *Chl*: *F*_1,7_ = 5.76, *p* = 0.0474, Fig. 3B), indicating that their values under combined high light and high temperature were higher than expected from the additive effects of each factor alone. In other words, the combined effect of high light and high temperature on *Chl*:*POC* and cell-specific *Chl* was not as strongly negative as expected from the sum of their individual effects, demonstrating an antagonistic interaction for these variables. Under future conditions in the Southern Ocean, cells may therefore maintain more *Chl* than expected, potentially sustaining higher light absorption capacity.

The photosynthetic efficiency parameters ${F}_{v}/{F}_{m}$ and *NPQ* showed similar responses to light and temperature. While temperature showed no effect on ${F}_{v}/{F}_{m}$, light had a statistically significant effect on the parameter, with lower values under high light compared with low light (Fig. 4B). Similarly, *NPQ* was significantly lower under high light than under low light at 8 °C (Fig. 4A). However, the magnitude of differences between treatments for ${F}_{v}/{F}_{m}$ and *NPQ* was minimal compared to other studies (Kulk et al. 2019; Pagnone et al. 2021), suggesting that despite statistical significance, these differences are unlikely to represent biologically meaningful changes in photosynthetic efficiency.

Both ${F}_{v}/{F}_{m}$and *NPQ* remained within a narrow range typically associated with non-stressed photosystems (${F}_{v}/{F}_{m}$: 0.472 ± 0.003 to 0.512 ± 0.003, Fig. 4B; *NPQ*: 0.85 ± 0.02 to 1.03 ± 0.02, Fig. 4A). This indicates limited photoinhibition under high light conditions (Parkhill et al. 2001) and minimal dissipation of excess excitation energy as heat (Trimborn et al. 2014). Other Southern Ocean diatom studies have found that ${F}_{v}/{F}_{m}$ is more sensitive to changes in iron concentrations than to changes in light (Moreno et al. 2018). In our study, the maximum quantum yield of PSII remained relatively high and stable, and *NPQ* remained low across treatments, consistent with other Southern Ocean diatoms grown in nutrient-replete conditions (Parkhill et al. 2001; Rose et al. 2009). While the high light conditions in our study (200 µmol photons m^−2^ s^−1^) represent a high value for an annual average light concentration in the Southern Ocean, it does not exceed periodic light exposure for phytoplankton in the region (Andrew et al. 2019). These results highlight the physiological flexibility of *P. subcurvata* photosystems, enabling cells to maintain high photosynthetic efficiency across a range of temperature and light conditions.

Importantly, *NPQ* and ${F}_{v}/{F}_{m}$ remained stable under combined high light and high temperature when chlorophyll concentrations were higher than expected in cells (Fig. 3A and B). Together, these findings suggest that under future conditions in the Southern Ocean, *P. subcurvata* cells may be able to improve their light absorption capacity while maintaining high photosynthetic efficiency, thus directing additional absorbed energy into photosynthesis and biomass production, ultimately leading to increased productivity. These physiological mechanisms help explain modelled projections of increased Southern Ocean productivity under future conditions (Bopp et al. 2013; Krumhardt et al. 2022; Leung et al. 2015; Moore et al. 2018).

### Implications for Southern Ocean ecosystems under future conditions

As climate change proceeds, the Antarctic zone of the Southern Ocean (south of 65°S) is projected to experience surface ocean warming and higher irradiances due to declining sea ice and shoaling mixed layers (Deppeler and Davidson 2017; Leung et al. 2015). Our findings suggest that diatoms, which are dominant south of the Polar Front (~60°S), may enhance their productivity under such conditions by adjusting their photosynthetic physiology. These results support projections of productivity increase in this region (Bopp et al. 2013; Fisher et al. 2025; Krumhardt et al. 2022; Leung et al. 2015; Moore et al. 2018) while highlighting key interactions between temperature and light driving changes in productivity (Boyd et al. 2016; Deppeler and Davidson 2017; Krumhardt et al. 2022). In addition to temperature and light interactions, incubation studies have also shown that *P. subcurvata* responds synergistically to increased temperature and iron availability (Zhu et al. 2016), underscoring the role of iron in likely contributing to the future increase in productivity in the Southern Ocean (Leung et al. 2015). Increases in primary productivity could have mixed effects on biogeochemical cycles in the Southern Ocean and other regions (Henley et al. 2020; Weber and Deutsch 2010). While greater primary productivity is likely to increase carbon export to the deep ocean (Cabré et al. 2014) and enhance nutrient uptake and recycling in the Southern Ocean (Moore et al. 2018), greater nutrient uptake in the Southern Ocean is also likely to decrease nutrient export northward in mode and intermediate waters (Marinov et al. 2006; Sarmiento et al. 2004), exacerbating declining productivity and carbon export in lower latitudes (Laufkötter et al. 2016; Moore et al. 2018).

Importantly, the projected increase in diatom productivity must be considered alongside shifts in Southern Ocean phytoplankton communities. Although sea ice retreat and greater light availability are expected to increase overall primary productivity, smaller phytoplankton such as cryptophytes are projected to increase more rapidly than diatoms because increasing stratification and freshening are intensifying iron limitation, disadvantaging the relatively iron-demanding diatoms (Deppeler and Davidson 2017; Fisher et al. 2025; Hayward et al. 2025; Krumhardt et al. 2022). Moreover, *P. subcurvata* seems to be better adapted to medium irradiances, as growth rates under both low and high light conditions in our study were lower than those reported in other studies (Kulk et al. 2019; Zhu et al. 2017). With *P. subcurvata*’s limited light optimum and reported low capacity to adapt to rapid changes in light conditions (Petrou et al. 2011), the species might lose out to cryptophytes that have rapid photoacclimation responses to increased irradiance (Ferreira et al. 2026). A shift from diatom-dominated communities to smaller phytoplankton could reduce biological carbon export and alter Antarctic food-web structure by replacing large, fast-sinking, krill-supporting diatoms with smaller taxa that are preferentially grazed by salps (Harbou et al. 2011), potentially decreasing carbon sequestration efficiency and the transfer of energy to higher trophic levels (Ballerini et al. 2014; Deppeler and Davidson 2017; Hayward et al. 2025; Johnston et al. 2022; Quéguiner 2013).

## Conclusion

The results of this study highlight important interactive effects between ocean warming and higher irradiances on *P. subcurvata* productivity and photosynthetic physiology. While elevated irradiances alone enhanced carbon and nitrogen production, only under warmer conditions did this translate into higher cell division rates. Moreover, ocean warming and higher irradiances interactively enhanced *Chl*:*POC* through two mechanisms: the dampening of light-induced *Chl* downregulation with warming, and enhanced *Chl* production due to warming under high light. Photosynthetic efficiency stayed high across treatments. The findings of this study suggest that in future Southern Ocean conditions, diatoms may increase their productivity by maintaining a relatively high light absorption capacity and light processing efficiency. These results help to elucidate poorly understood physiological mechanisms driving changes in primary productivity while also highlighting the essential role of incubation studies in understanding interactive effects between environmental factors on phytoplankton physiology and productivity. Future research should include larger sample sizes, which were limited to 14 in this study by incubator space constraints, to improve the precision of parameter estimates. Extending similar experiments to other Southern Ocean diatom species would also help determine whether the physiological responses observed here are consistent between species. Ultimately, incorporating the interactive responses of growth and photosynthetic physiology to warming and higher irradiances into primary productivity models could improve the accuracy of future projections of Southern Ocean primary productivity as climate change proceeds.

## Supplementary Information

Below is the link to the electronic supplementary material.

Supplementary Material 1

## Data Availability

All raw data supporting this research are available in the following repository: https://github.com/olegouvello/Interactive_effects_Pseudo_nitzschia_data.
