# Supplementary material for: Interactive effects of ocean warming and higher irradiances on the Southern Ocean diatom Pseudo-nitzschia subcurvata
Source: Polar Biol. 2026 Oct 10;49(4):116. doi: 10.1007/s00300-026-03542-6 (PMC13653955; doi:10.1007/s00300-026-03542-6)
Supplement: Supplementary file 1 [file 300_2026_3542_MOESM1_ESM.pdf]

Supplementary Information

Article title: Interactive effects of ocean warming and higher irradiances on the Southern Ocean diatom *Pseudo-nitzschia subcurvata*

Journal name: Polar Biology

Olivia Le Gouvello[1], Ben J. Fisher[1,2], Sian F. Henley[1]

[1] School of GeoSciences, University of Edinburgh, Edinburgh, United Kingdom

[2] Lyell Centre for Earth and Marine Science and Technology, Heriot-Watt University, Edinburgh, United Kingdom

Corresponding author: Olivia Le Gouvello (olivia.legouvello@gmail.com)

Table S1 Volumes filtered for Particulate Organic Carbon (POC), Particulate Organic Nitrogen (PON) and chlorophyll a (Chl) analysis on day 11. Smaller volumes were filtered for culture bottles with higher cell densities (e.g., high light, high temperature). Smaller volumes were also filtered for controls due to the large amount of cytoplasmic solute slowing filtration.

**Treatment**	**Sample number**	**Volume filtered for POC and PON analysis (mL)**	**Volume filtered for Chl analysis (mL)**
Low light low temperature	1	200	150
	2	200	150
	3	200	150
High light low temperature	4	100	100
	5	100	100
	6	100	100
No light low temperature (control)	7	200	100
Low light high temperature	8	150	150
	9	150	150
	10	150	150
High light high temperature	11	80	80
	12	80	80
	13	80	80
No light high temperature (control)	14	125	125

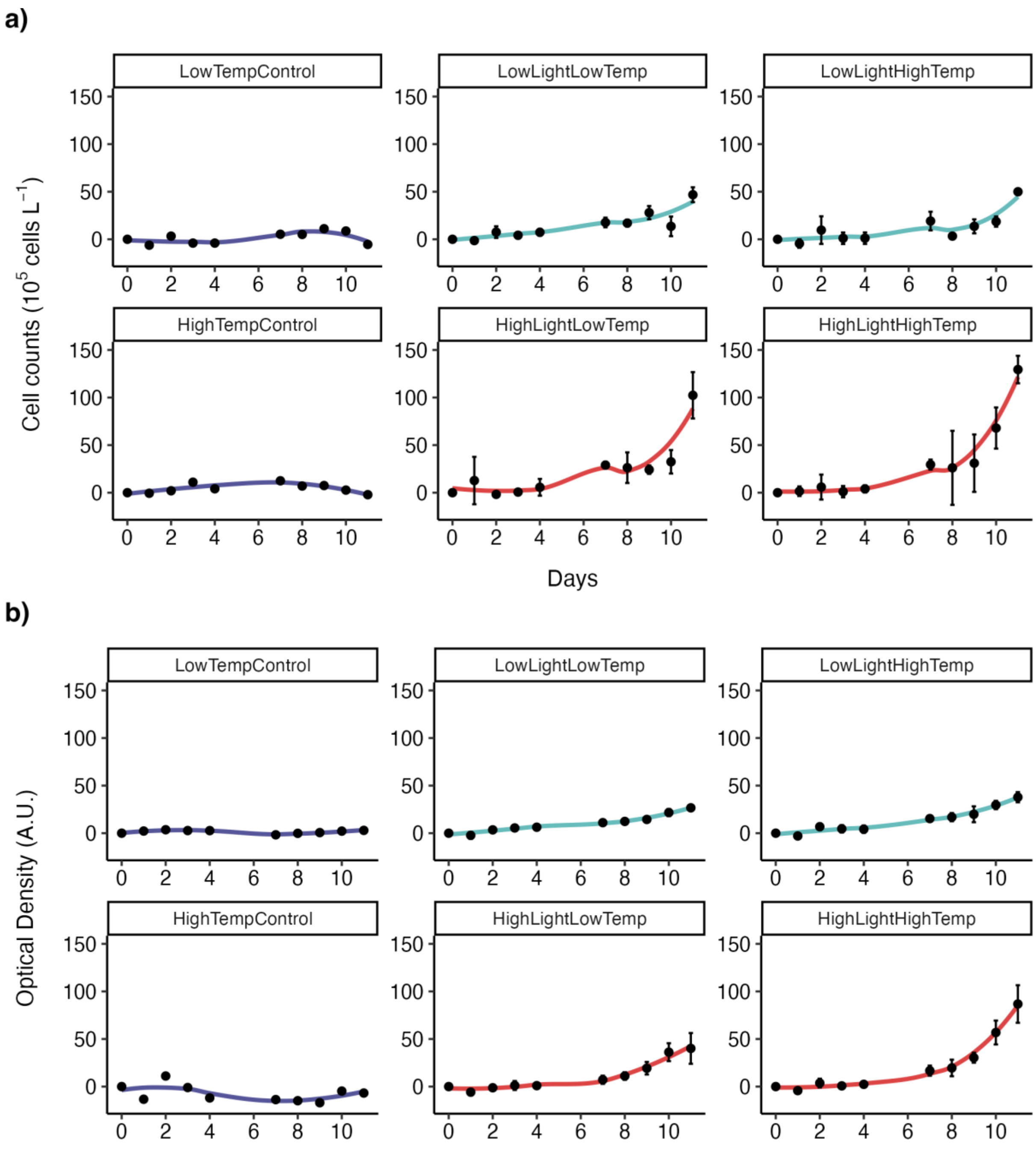

Fig. S1 Cell counts (10^5 cells μL^{-1}) (a) and optical density (A.U. day^{-1}) (b) over time in each treatment**.** Cell counts and optical density were normalised to day 0, where values on day 0 were standardised to 0. Subsequent day values represent the difference between the cell counts or optical density on that day and the respective values on day 0. Error bars represent the standard error of the mean cell count each day (n = 3).

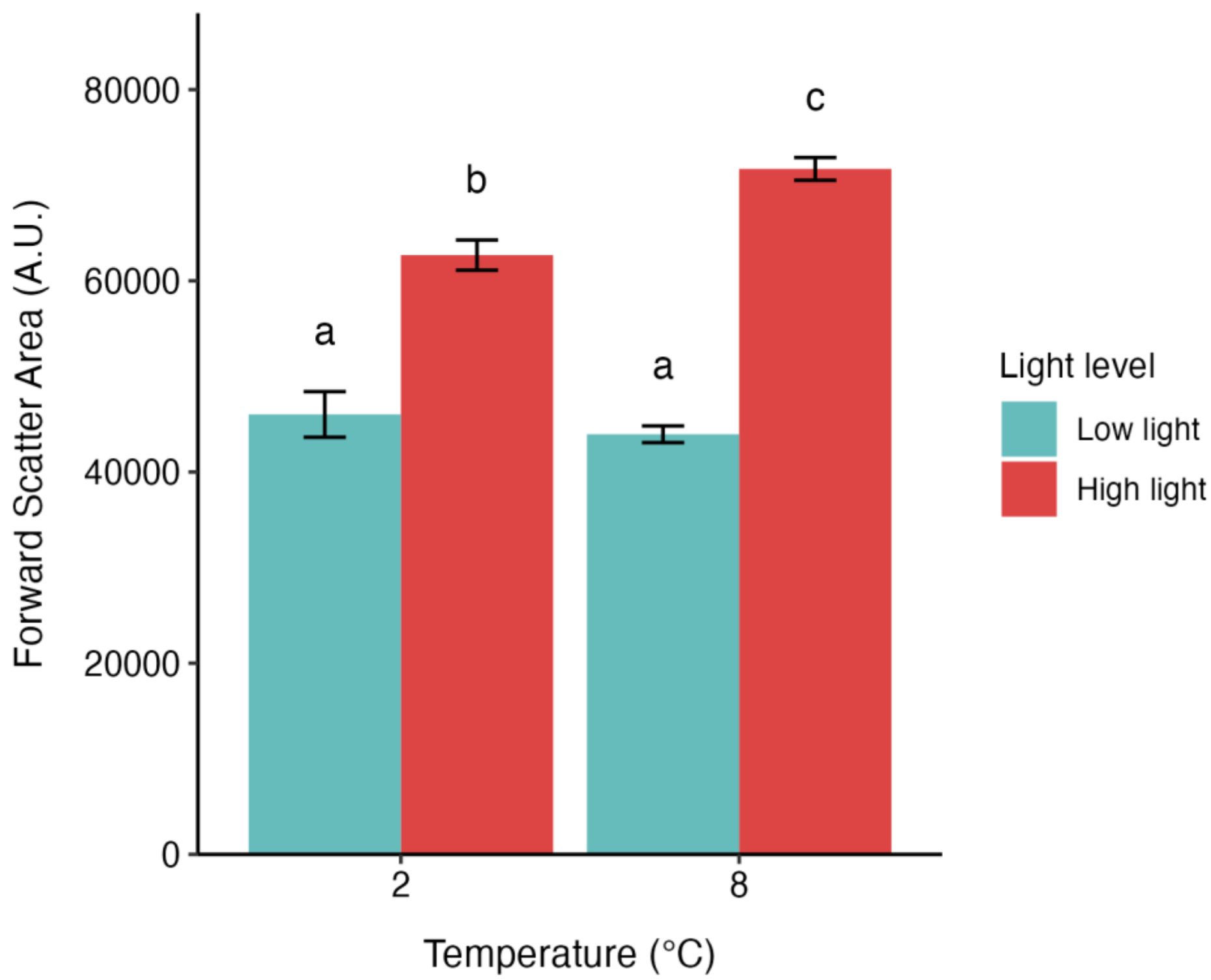

Fig. S2 Forward Scatter Area (in arbitrary units, A.U.) for each treatment. Forward Scatter Area data was derived from the Flow Cytometry measurements and acts as a proxy for cell size. Error bars represent standard errors of the means for each treatment ($n = 3$) and different letters indicate significant differences between treatments (i.e., a is significantly different to b).
